# Antithrombotic Treatment After Surgical and Transcatheter Heart Valve Repair and Replacement

**DOI:** 10.3389/fcvm.2021.702780

**Published:** 2021-08-05

**Authors:** Andreas Verstraete, Marie Christine Herregods, Peter Verbrugghe, Marie Lamberigts, Thomas Vanassche, Bart Meyns, Wouter Oosterlinck, Filip Rega, Tom Adriaenssens, Lucas Van Hoof, Siegmund Keuleers, Christophe Vandenbriele, Peter Sinnaeve, Stefan Janssens, Christophe Dubois, Bart Meuris, Peter Verhamme

**Affiliations:** ^1^Department of Cardiovascular Diseases, University Hospitals Leuven, Leuven, Belgium; ^2^Department of Cardiac Surgery, University Hospitals Leuven, Leuven, Belgium

**Keywords:** anTithrombotic treatment, valve intervention, surgical valve replacement, surgical valve repair, transcatheter valve repair, transcatheter valve replacement, transcatheter aortic valve implantation, valvular heart disease

## Abstract

New antithrombotic drugs have been developed, new valve types have been designed and minimally invasive transcatheter techniques have emerged, making the choice of antithrombotic therapy after surgical or transcatheter heart valve repair and replacement increasingly complex. Moreover, due to a lack of large randomized controlled trials many recommendations for antithrombotic therapy are based on expert opinion, reflected by divergent recommendations in current guidelines. Therefore, decision-making in clinical practice regarding antithrombotic therapy for prosthetic heart valves is difficult, potentially resulting in sub-optimal patient treatment. This article compares the 2017 ESC/EACTS and 2020 ACC/AHA guidelines on the management of valvular heart disease and summarizes the available evidence. Finally, we established a convenient consensus on antithrombotic therapy after valve interventions based on over 800 annual cases of surgical and transcatheter heart valve repair and replacement and a multidisciplinary team discussion between the department of cardiovascular diseases and cardiac surgery of the University Hospitals Leuven, Belgium.

## Introduction

Valvular heart disease is a major and growing challenge worldwide and for severe valvular heart disease, valve intervention is the only effective treatment. Nowadays, ~300 000 artificial valves are implanted every year and it is estimated that this number will further increase (1).

Heart valve surgery improves survival, morbidity and the quality of life (1). Heart valve repair is the preferred strategy in regurgant heart valves and results in a life expectancy similar to the general population (2, 3). But not all valves can be repaired and in these cases, valve replacement represents a valid alternative. However, a valve prosthesis introduces new risks into patients' lives, translating into a worse relative survival compared to the general population (4, 5). Artificial valves require antithrombotic treatment for a limited or unlimited duration and consequently, patients are exposed to an increased risk of bleeding. Over the past decades substantial technological progress has been made in the field of valvular treatment: transcatheter techniques have arisen, repair techniques and valve designs have been optimized and new antithrombotic drugs have been developed. To date, three categories of prosthetic heart valves can be distinguished: surgical mechanical valves, surgical bioprosthetic valves and transcatheter (bioprosthetic) valves. The thromboembolic risk is determined by patient characteristics and the type and position of the prosthetic valve. Due to lower flow and pressure conditions, the risk of thrombotic complications is higher with right-sided prosthetic valves. Similarly, hemodynamic differences result in a higher thrombotic risk for mitral valves compared to aortic valves, reflected by the different historical INR target ranges for the various valve positions (6). Defining an optimal antithrombotic therapy for patients with these different valve types is crucial to minimize the risk for associated thromboembolic and bleeding complications.

The aim of this article is to evaluate the 2017 ESC/EACTS and 2020 ACC/AHA guidelines for the management of valvular heart disease and formulate a convenient and unambiguous consensus on antithrombotic treatment for prosthetic heart valves and valve repair at the University Hospitals Leuven, Belgium, based on the available literature and expertise.

## Materials and Methods

Starting from an in-depth analysis of the 2017 ESC/EACTS and 2020 ACC/AHA guidelines for the management of valvular heart disease, we conducted a comprehensive literature search of PubMed, the Cochrane Library, and ClinicalTrials.gov from inception to December 2020 (7, 8). We used several combinations of key words relating to valve repair or replacement (“heart valves” [MeSH], “heart valve prosthesis” [MeSH], “bioprosthesis” [MeSH], “mechanical heart valve,” “transcatheter aortic valve replacement” [MeSH], “transcatheter mitral valve replacement, “MitraClip,” “transcatheter pulmonary valve replacement,” “transcatheter tricuspid valve replacement,” “surgical valve repair”) and key words relating to antithrombotic treatment (“anticoagulants” [MeSH], “anticoagulation,” “antithrombins” [MeSH], “antithrombotic,” “coumarins” [MeSH], “platelet aggregation inhibitors” [MeSH], “factor Xa inhibitors” [MeSH], thrombotic risk) to enhance the search sensitivity. Another search term was “subclinical leaflet thrombosis.” Included articles were restricted to those in English that evaluated antithrombotic therapy after surgical or transcatheter heart valve repair or replacement. All articles were included according to a two-step procedure. First a selection was made after reviewing title and abstract. Subsequently, the remaining articles were subjected to a full-text screening. Finally, the references of the retrieved papers were searched to identify additional, potentially relevant articles.

After a critical appraisal of the guidelines and available evidence, a multidisciplinary discussion took place involving physicians of the heart valve and the thrombosis care program of the department of cardiovascular diseases and cardiac surgery of the University Hospitals Leuven, Belgium. Based on this team approach on 833 cases in 2019, we formulated a practical local consensus for antithrombotic therapy in patients undergoing heart valve replacement or repair, which can serve as a practical guidance for decision-making in daily clinical practice.

## Results

### Mechanical Valves

Due to an inherent thrombogenicity, life-long oral anticoagulation (OAC) with vitamin K antagonists (VKA) is currently indicated after mechanical valve implantation. In a meta-analysis, Cannegieter et al. demonstrated a 4-fold increase of the thromboembolic risk in the absence of VKAs (9). The risk for major complications in patients with a mechanical heart valve varies across different studies. The rate of thromboembolic complications ranges from <0.1% per year to 2.75% per year and the rate of bleeding from 0.24% per year to 3.31% per year (10). In a meta-analysis of non-elderly patients with a mechanical aortic valve, the pooled rate of thromboembolism and major bleeding were 0.90% per year and 0.85% per year, respectively (11). In a retrospective analysis, the cumulative incidence of stroke at 15 years follow-up was 8.6% after aortic valve replacement and 14% after mitral valve replacement (12, 13). The 15-year cumulative incidence of major bleeding was 13 and 15% after aortic and mitral valve replacement, respectively (12, 13).

#### Vitamin K Antagonists

The reduced thrombogenicity of current generation bileaflet mechanical prostheses allows a lower target INR than traditionally pursued with older generation mechanical valves. To date, American and European guidelines have reached consensus on the optimal INR values for current generation bileaflet mechanical aortic and mitral valves (Table 1). In patients without thromboembolic risk factors an INR of 2.5 and 3.0 should be targeted for the aortic and mitral position, respectively (7, 8). Common cited thromboembolic risk factors are atrial fibrillation, previous thromboembolism, left ventricle dysfunction and hypercoagulability (14–16). In the presence of any of these risk factors, it is recommended that the target INR is increased to 3.0, regardless of the valve position. Tricuspid valve replacement is rare and most often performed during left-sided valve surgery when valve repair is not feasible. In patients with a mechanical tricuspid prosthesis, the ESC and EACTS recommend a target INR of 3.0 (7).

**Table 1:** Overview and comparison of 2017 ESC/EACTS and 2020 ACC/AHA guidelines for the management of valvular heart disease.

	**ESC/EACTS 2017** ^**a**^		**ACC/AHA 2020** ^**a**^	
	**Aortic**	**Mitral/Tricuspid**	**Aortic**	**Mitral**
**Mechanical valves**				
Bileaflet - ATS - Carbomedics - Medtronic - On-X - St. Jude Medical - Sorin Bicarbon	INR 2.5^b^	INR 3.0^b^	INR 2.5^b^	INR 3.0^b^
+ Risk factors^c^	INR 3.0^b^	INR 3.0^b^	INR 3.0^b^	INR 3.0^b^
On-X Valve (after 3 m)	No guidelines	No guidelines	INR 1.5–2.0 +ASA^d^	No guidelines
Older-Generation: - Björk-Shiley - Lillehei-Kaster - Omniscience - Starr-Edwards	INR 3.5^b^	INR 4.0^b^	INR 3.0^b^	INR 3.0^b^
+ Risk factors^c^	INR 4.0^b^	INR 4.0^b^	INR 3.0^b^	INR 3.0^b^
+ Thromboembolism^e^	+ ASA	+ ASA	+ ASA	+ ASA
**Bioprosthetic valves**				
Without indication for OAC				
Early (<3–6 months)	ASA (3 m) or INR 2.5 (3 m)	INR 2.5 (3 m)	ASA or INR 2.5 (3–6 m)	ASA or INR 2.5 (3–6 m)
Late (>3–6 months)	No therapy required	No therapy required	ASA	ASA
With indication for OAC	INR 2.5 (3 m)	INR 2.5 (3 m)	INR 2.5 (3–6 m)	INR 2.5 (3–6 m)
**Transcatheter valves**				
Without indication for OAC				
Early (<3–6 months)	ASA or DAPT	No guidelines	ASA or DAPT or INR 2.5	No guidelines
Late (>3–6 months)	ASA		ASA	
With indication for OAC	INR 2.5 (3–6 m)		INR 2.5 (3–6 m)	
**Surgical Valve Repair** ^f^				
	ASA (3 m)	INR 2.5 (3 m)	No guidelines	No guidelines

a *ASA dose: 75–100 mg/day; INR refers to recommended use of VKA*.

b *ASA addition can be considered in case of concomitant atherosclerotic disease or another indication for antiplatelet therapy*.

c *Risk factors: atrial fibrillation, previous thromboembolism, left ventricle dysfunction or hypercoagulability*.

d *From 3 months after On-X aortic valve replacement, an INR of 1.5 to 2.0 can be targeted in conjunction with ASA*.

e *In case of thromboembolism despite an adequate INR, ASA is associated*.

f *The ESC/EACTS for antithrombotic treatment after surgical valve repair are restricted to the early post-operative phase in patients without an OAC indication. The 2020 ACC/AHA do not provide recommendations for surgical valve repair*.

*ACC, American College of Cardiology; AHA, American Heart Association; ASA, acetyl salicylic acid; DAPT, dual antiplatelet therapy; ESC, European Society of Cardiology; EACTS, European Association for Cardiothoracic Surgery; INR, International Normalized Ratio; OAC, oral anticoagulation*.

Recent studies argue for a further reduction of the INR in well-selected patient populations. In a randomized controlled trial in low-risk patients with a solitary bileaflet mechanical aortic valve (>70% Sorin Bicarbon and 22% St. Jude Medical), Torella et al. demonstrated the benefit of a lower target INR of 2.0: less bleeding complications (3 vs. 8%, OR 0.36) while being equally effective in terms of thrombotic risk (14). Moreover, the PROACT trial showed that from 3 months after an On-X aortic valve replacement, a lower target INR of 1.5 to 2.0 in combination with low-dose aspirin, can be considered, as already included in the American guidelines (17). In addition, the PROACT trial evaluated a dual antiplatelet strategy with aspirin and clopidogrel after On-X aortic valve replacement. However, dual antiplatelet therapy (DAPT) significantly increased the number of thromboembolic complications and thus is not recommended (17).

A stable INR is of the utmost importance as fluctuating INR values increase the risk of thrombotic and hemorrhagic complications (18). Early INR self-management after mechanical valve replacement improves the time in therapeutic range and significantly reduces anticoagulation-related complications compared to INR management by the physician (18). Furthermore, INR self-management can contribute to a further decline of the target INR. The ESCAT III trial revealed that, from 6 months after aortic valve replacement, an INR of 1.6 to 2.0 in the setting of weekly INR self-management under the watchful eye of a controlling physician did not result in an excess of thrombotic events, and reduced bleeding risk (0.67 vs. 1.93% per patient-year, HR 0.38) (19). Hence, where feasible, point of care INR self-management should be offered to selected patients through better quality of INR control, lower morbidity and better quality of life and patient satisfaction (7, 20).

#### Addition of Aspirin

Until recently, the systematic addition of low-dose aspirin to VKAs was a major point of controversy between European and American guidelines. However, the 2020 ACC/AHA guidelines no longer recommend the systematic addition of low-dose aspirin in patients with mechanical valves (8). According to the 2020 ACC/AHA and 2017 ESC/EACTS guidelines, aspirin association may be considered in the presence of a concomitant indication for antiplatelet therapy or in case of thromboembolism despite an adequate INR (7, 8). It is difficult to compare individual studies about the benefit of aspirin association because of marked heterogeneity with respect to type and dose of the anti-platelet drugs. Older studies seem to favor the combination and report reduced thromboembolic complications without a significant increase in bleeding risk (21, 22). More recent studies show a more nuanced picture with a clear decrease in thromboembolic risk (OR 0.27–0.33) at the expense of a significant increase in hemorrhagic events (OR 1.49–2.19) (23–25). The consensus of the panel members of this guidance document was therefore to not systematically add low-dose aspirin.

#### Role of Non-vitamin K Oral Anticoagulants

Non-Vitamin K oral anticoagulants (NOACs) have major advantages over VKAs due to an improved safety profile as they reduce the risk of intracranial hemorrhage and obviate the need for routine INR monitoring. They have proven to be effective in the context of atrial fibrillation, deep vein thrombosis and pulmonary embolism. On the other hand, all NOACs remain contraindicated in the setting of mechanical valve prostheses as the only trial with this class was unfavorable (15). The RE-ALIGN study evaluated the use of dabigatran, a direct thrombin (factor IIa) inhibitor, in patients with mechanical heart valves, but was discontinued prematurely because of excess bleeding (27 vs. 12%) and thromboembolic events (9 vs. 5%) in patients treated with dabigatran compared to warfarin (15). However, certain aspects of this study need to be highlighted in order to possibly consider a role for NOACs in the future. First, optimal dabigatran dose regimen in the indication of mechanical valve prostheses remains to be established since a significant proportion of patients showed lower trough plasma levels of dabigatran during the 1st weeks after surgery (15). Second, the role of factor Xa inhibitors in patients with a mechanical heart valve has never been investigated, and their pharmacodynamics clearly differ from factor IIa inhibitors. Clotting on mechanical valves is partially mediated via the contact pathway by activation of tissue factor which generates large amounts of thrombin, potentially overwhelming the local concentration of factor IIa inhibitors. Factor Xa inhibitors inhibit the coagulation cascade more upstream to thrombin and may thus be more effective as one molecule of factor Xa activates more than thousand molecules of thrombin. However, efficacy and safety of factor Xa inhibitors in patients with mechanical heart valves remain to be studied in adequate clinical trials. A long-awaited study is currently ongoing: the prospective, randomized PROACT Xa trial is evaluating the safety and efficacy of apixaban from 3 months after On-X aortic valve replacement and will provide important information on a possible future role of NOACs in mechanical valves (26). Alternatively, future perspectives in anticoagulation in mechanical heart valves may include the use of a NOAC in combination with specific inhibitors of the contact pathway, such as factor XIa-inhibitors. Such molecules are currently under development, and their place and role remain to be investigated.

### Bioprosthetic Valves

The field of the bioprosthetic valves is evolving. New bioprosthetic valves are now increasingly implanted in younger patients to avoid anticoagulation and related complications. This trend is supported by an improved durability of bioprostheses and because valve degeneration no longer mandates major redo surgery as re-intervention is increasingly performed percutaneously using transcatheter valve techniques.

The thromboembolic risk of bioprosthetic valves is greatest in the first 10 days after surgery and gradually decreases thereafter through progressive endothelialisation of the valve surface (27). After 90 days, endothelialisation is complete, resulting in a similar thromboembolic risk in patients with or without antithrombotic therapy (27). Consequently, most physicians prescribe antithrombotic drugs for at least 3 months. Nevertheless, there is no consensus on the duration of antithrombotic treatment and further research is needed to define the optimal time window. The ESC and EACTS recommend a 3-month period of antithrombotic therapy, while ACC/AHA guidelines extend this treatment up to 6 months after bioprosthetic valve implantation (7, 28).

#### Early Post-operative Phase

The use of VKAs has long been the gold standard for antithrombotic therapy in the 1st months after placement of a bioprosthetic valve. Currently, the ESC/EACTS guidelines recommend VKAs with a target INR of 2.5 in all patients with a mitral or tricuspid bioprosthesis (7). In patients with a bioprosthetic aortic valve and no other indication for OAC, European guidelines prefer aspirin monotherapy, although VKAs (target INR 2.5) can be an alternative. Already in 2004, a prospective study by Gherli et al. showed equivalence between low-dose aspirin and warfarin with respect to ischemic and bleeding complications during the first 3 months after bioprosthetic aortic valve replacement (29). Since then, several studies have confirmed these findings and, in 2019, a meta-analysis concluded that antiplatelet monotherapy in the first months after bioprosthetic aortic valve implantation had a more beneficial efficacy and safety profile due to a lower bleeding risk (OR 0.32) (30).

In the ACC/AHA guidelines, aspirin monotherapy can be used as an alternative to VKAs (target INR 2.5) after both bioprosthetic aortic or mitral valve implantation (28). However, the recommendation for aspirin monotherapy after bioprosthetic mitral valve replacement is an extrapolation of the findings in bioprosthetic aortic valves and has never been investigated. While awaiting a prospective study comparing low-dose aspirin with VKAs after bioprosthetic mitral valve replacement, the consensus was to reserve low-dose aspirin monotherapy in the early post-operative phase for patients with a bioprosthetic aortic valve at low thromboembolic risk. Deep vein thrombosis, pulmonary embolism, hypercoagulability, atrial fibrillation, a reduced left ventricular function and mitral or tricuspid valve replacement are indications to start VKAs anyway.

#### Late Post-operative Phase

In the late phase, from 3 to 6 months after surgery, there is controversy about the need for lifelong low-dose aspirin. American guidelines support lifelong low-dose aspirin administration in patients without OAC indication (8). According to European guidelines, it is only appropriate in the case of concomitant indications for antiplatelet therapy (7). Little or no literature exists on the advantages or disadvantages of lifelong aspirin administration after bioprosthetic valve implantation, which explains the divergent guidelines. The decision is therefore a personal preference of the physician based on local consensus and experience rather than being based on hard evidence.

#### Role of NOACs

Until recently, little was known about the use of NOACs in bioprosthetic valves. Small studies suggested that NOACs could be safely used to treat atrial fibrillation from 3 months after bioprosthetic valve implantation (31, 32). This seems plausible as gradual endothelialisation of the prosthetic valve results in a more or less physiological state after 3 months. Recently, a larger randomized controlled trial on this subject confirmed these findings (33). The RIVER trial showed that in patients with a mitral bioprosthesis and atrial fibrillation or flutter, rivaroxaban (20 mg/day) was not inferior to warfarin (INR 2.0 to 3.0) with respect to the composite of death, major cardiovascular events and major bleeding at 12 months follow-up (33). Furthermore, it was the first time that the use of NOACs in the early phase after surgery was investigated, as 18.8% of 1,005 patients underwent randomization within 3 months after mitral valve surgery. Although this subgroup analysis should be interpreted with caution, the results were consistent. Future studies are needed to fully clarify the role of NOACs in bioprostheses, especially in the early phase after surgery.

### Surgical Valve Repair

Notwithstanding the wide range of prosthetic heart valves and the significant improvements in this field over the last decades, valve repair remains the first-choice surgical treatment for severe valvular heart disease when technically feasible as it preserves the native valve apparatus and does not require lifelong OAC.

Since many years, mitral and tricuspid valve repair are established techniques. Tricuspid valve repair is usually performed during left-sided valve surgery. Isolated tricuspid valve (repair) surgery is uncommon, and associated with a high operative mortality of 8.8% (7, 34). Recently, surgical aortic valve repair has also been performed more frequently, and if not possible, a Ross operation can be considered until the age of 60. Although technically demanding, long-term outcomes after aortic valve repair in experienced centers are good (35). Moreover, perioperative outcomes are comparable to aortic valve replacement, with a trend toward a lower 1-year mortality, despite a higher re-intervention rate at 1 year (36). Surgical aortic valve repair can therefore be considered in well-selected patients in high volume, experienced centers.

The current 2020 ACC/AHA guidelines provide no recommendations for the antithrombotic treatment after surgical valve repair. However, both the ESC/EACTS and 2017 AHA/ACC guidelines recommend thromboprophylaxis with VKAs (INR 2.0–3.0) during the first 3 months after mitral valve repair (7, 28). In addition, the ESC/EACTS recommend 3 months of anticoagulation with VKAs (INR 2.0–3.0) for repaired tricuspid valves (7). After aortic valve repair, low-dose aspirin monotherapy can be considered provided that thromboembolic risk factors are absent (7). The evidence for antithrombotic drugs after surgical valve repair is limited. Guidelines are mainly based on expert opinion, but the use of an annuloplasty ring and the possible occurrence of post-operative atrial fibrillation can justify short-term antithrombotic therapy. Observational studies suggest that VKAs are not superior to low-dose aspirin regarding the thromboembolic prevention after mitral valve repair, but potentially increase the risk of bleeding (37, 38). Furthermore, NOACs appear to be a potential alternative, but more data validating NOACs are needed (39). So far, an antithrombotic strategy after aortic valve repair has not been studied as the technique is in full development. Larger studies and randomized controlled trials are necessary to justify the need for thromboprophylaxis after surgical valve repair and to identify a well-founded antithrombotic policy.

### Transcatheter Aortic Valve Implantation

As the prevalence of severe aortic stenosis increases with age, there is a growing interest in minimally invasive valve replacement techniques for fragile patients with multiple co-morbidities who are not suitable for surgery. After two decades of technological refinement, TAVI is now an established treatment for patients with severe symptomatic aortic stenosis at high or intermediate surgical risk (7, 40–42). Recent studies have also shown that TAVI is a valid alternative for surgical aortic valve replacement in low risk groups (43).

As with surgical prosthetic heart valves, thromboembolic and bleeding complications are an important concern after TAVI and uncertainty regarding the optimal antithrombotic strategy remains. Despite antithrombotic treatment with DAPT or OAC, the incidence of stroke remained 4.1 and 7.0% at 30 days and 1 year after the procedure, respectively (44). The rate of major bleeding amounts to 10.2% during the first 30 days and rises to 16.0% in the 1st year, reflecting the frailty of TAVI patients (44).

Many TAVI patients have an indication for longstanding OAC. Approximately 40% of patients have associated atrial fibrillation and up to 15% develop new-onset atrial fibrillation after TAVI (44). Consequently, a stratification according to the need for OAC is recommended in the choice of antithrombotic therapy.

#### Without Indication for Oral Anticoagulation

In patients without an indication for OAC, the ESC/EACTS and ACC/AHA guidelines originally recommended systematic DAPT with aspirin and clopidogrel for 3 to 6 months after TAVI, followed by lifelong low-dose aspirin (7, 8). However, the ARTE randomized controlled trial showed that single antiplatelet therapy with low-dose aspirin reduced the risk of major bleeding (3.6 vs. 10.8%, OR 0.31) without increasing the incidence of stroke, myocardial infarction and mortality (45). Recently, cohort A of the POPular-TAVI trial convincingly demonstrated a reduction of bleeding (15.1 vs. 26.6%, OR 0.49) and the composite of bleeding and thromboembolic events in patients treated with low-dose aspirin monotherapy as compared with DAPT in this setting, which support the recommendation for aspirin monotherapy as an alternative to DAPT in the actual ESC/EACTS and ACC/AHA guidelines (46).

Additionally, the 2020 ACC/AHA guidelines also recommend VKAs with a target INR of 2.5 in TAVI patients at low bleeding risk without an indication for chronic OAC (28). This recommendation is based on imaging studies showing an incidence of subclinical valve thrombosis after TAVI of ~15%, and an observational study by Chakravarty et al. showing the potential, preventive effect of OAC in this context (47, 48). However, little is known about the use of OAC in TAVI patients without a firm indication for OAC. The comparison of low-dose rivaroxaban (10 mg/day) (in association with low-dose aspirin during the first 3 months) with DAPT in the randomized GALILEO trial resulted in a higher incidence of bleeding (5.6 vs. 3.8%, HR 1.50) and thromboembolic complications (12.7 vs. 9.5%, HR 1.35) and a higher mortality (7.7 vs. 4.6%, HR 1.69) with rivaroxaban, which explains the contraindication for NOAC in the 2020 ACC/AHA guidelines (49).

In summary, in absence of a firm indication for OAC, a single antiplatelet regimen with low-dose aspirin is preferred. The potential added value of OAC with either VKAs or NOACs to prevent (subclinical) thromboembolic events remains to be determined and weighed against the bleeding risk.

#### With Indication for Oral Anticoagulation

In patients with a concomitant indication for long-term OAC such as atrial fibrillation, venous thromboembolism or hypercoagulability, OAC should be continued post TAVI (7, 8). Recent evidence from cohort B of the POPular-TAVI trial convincingly showed that, in comparison to OAC plus 3 months clopidogrel, OAC monotherapy was associated with less bleeding complications (21.7 vs. 34.6%, OR 0.52) without an increase in ischemic stroke, myocardial infarction or cardiovascular mortality (50). OAC monotherapy can thus be continued after TAVI without the addition of antiplatelet agents, unless the latter are indicated for another reason (e.g., recent coronary stenting). Both VKAs and NOACs can be used in this setting according to standard dosing and practice guidelines. Of note, a recent prospective, observational study in patients with atrial fibrillation after TAVI reported that, in comparison with VKAs, NOAC therapy resulted in a significant reduction of all-cause mortality (10.3 vs. 23.3%, HR 0.39) (51). NOACs might potentially provide a survival benefit for patients with an OAC indication undergoing TAVI, but these findings need to be confirmed in a large randomized controlled trial.

#### Role of NOACs: Ongoing Trials

Several alternative treatment algorithms are currently being evaluated to assess the role of apixaban and edoxaban as alternative to VKAs in the setting of TAVI (52, 53). These ongoing trials will further extend our knowledge and validate future practical recommendations about NOACs in TAVI patients.

### Other Transcatheter Techniques: Percutaneous Edge-to-Edge Mitral Repair and Future Perspective

#### Edge-to-Edge Mitral Repair (MitraClip™)

Besides TAVI, percutaneous edge-to-edge repair with MitraClip™ has now become an established technique in transcatheter heart valve interventions. The EVEREST II and the COAPT trial demonstrated the safety and efficacy of transcatheter mitral valve repair with MitraClip™ in well-selected patients with moderate or severe mitral regurgitation (54, 55). So far, the antithrombotic policy after MitraClip™ has been based on expertise. In the EVEREST II trial, patients receiving MitraClip™ were treated with aspirin, 325 mg/day, for 6 months and clopidogrel, 75 mg/day, for 30 days (54). In the COAPT trial, standard regimen included aspirin, 81 mg/day, and/or clopidogrel, 75 mg/day, for 6 months or longer (55). In patients with chronic OAC, OAC was continued without the systematic addition of antiplatelet therapy (55).

#### Future Perspective

Since many patients with valvular heart disease are old and frail, and therefore ineligible for surgery due to a high or prohibitive surgical risk, numerous other transcatheter valve repair and replacement techniques are under development to address this unmet clinical need. Transcatheter mitral valve replacement is an emerging technology. Furthermore, more and more preliminary data are becoming available on transcatheter valve repair and replacement in tricuspid and pulmonary position. Until now, the antithrombotic policy for all these innovative techniques has been based on local expertise. In the absence of clear recommendations, it seems appropriate to follow antithrombotic regimens that are similar to the surgical technique that these approaches are mimicking. Future research should focus on optimal tailored antithrombotic treatments for new transcatheter procedures.

## Discussion

Antithrombotic treatment in patients with a prosthetic heart valve is complex and heterogeneous, given the diversity in patient characteristics, the wide range of prosthetic heart valves, the large arsenal of antithrombotic drugs and the lack of uniformity between studies. There is a paucity of large randomized controlled trials and many antithrombotic recommendations are based on expert opinions and non-prospective, observational studies. The lack of hard evidence is illustrated by the divergent recommendations between current ESC/EACTS and ACC/AHA guidelines for the management of valvular heart disease. Consequently, decision-making in clinical practice regarding antithrombotic therapy for prosthetic heart valves is difficult and not unequivocal, which may result in sub-optimal patient treatment and more thromboembolic and bleeding complications.

Following a multidisciplinary team approach involving physicians of the heart valve and the thrombosis care program of the department of cardiovascular diseases and cardiac surgery of the University Hospitals Leuven, Belgium, a consensus practical guidance was established for the antithrombotic treatment of patients undergoing heart valve replacement or repair. We have tried to formulate an unambiguous recommendation which can serve as a practical manual for decision-making in daily clinical practice (Table 2). The consensus applies to the prosthetic valves that are currently implanted and have been used in recent years. Because few patients remain with early generation heart valves (Table 1), these valves were not included in the contemporary overview table.

**Table 2:** University Hospitals Leuven consensus on antithrombotic treatment after heart valve repair and replacement^a^^,^ ^b^.

	**Aortic**	**Mitral/Tricuspid**
**Mechanical valves**		
Bileaflet	Target: INR 2.0–2.5 Window: INR 1.8–3.0^c^	Target: INR 2.5–3.0 Window: INR 2.0–3.5^c^
+ Thromboembolism^d^	+ ASA	+ ASA
**Bioprosthetic valves**		
Without OAC indication	ASA^e^	INR 2.0–3.0 (90 days)^f^, followed by ASA^e^
With OAC indication	NOAC^g^	NOAC^g^
**Surgical valve repair**		
Without OAC indication	ASA (90 days)	ASA
With OAC indication	NOAC^g^	NOAC^g^
**Transcatheter valve replacement/repair**	TAVI	MitraClip™
Without OAC indication	ASA	DAPT (30 days), followed by ASA
With OAC indication	NOAC^g^	ASA (30 days) + NOAC^g^

a *Overview of the recommendations on antithrombotic treatment for surgical and transcatheter heart valve repair and replacement in the University Hospitals Leuven (UZ Leuven), Belgium*.

b *ASA: low-dose aspirin (75–100 mg daily)*.

c *For mechanical valves two INR ranges are shown: a narrow, target INR range and a wider INR window. When a patient's INR is determined, we accept all values within the wide INR window. Dose adjustments are not necessary as long as the INR is within the INR window. In case of thrombo-embolic risk factors, an increase of the target INR by 0.5 may be considered individually, but the broader INR window remains unaffected*.

d *In case of thromboembolic events under VKA therapy, ASA is associated*.

e *Discontinuation of ASA can be considered in patients at high bleeding risk from 3 months after surgery*.

f *After bioprosthetic mitral or tricuspid valve replacement in patients without OAC indication, NOACs can be occasionally used as an alternative to VKAs, although not reimbursed in this context*.

g *VKAs (INR 2.0–3.0) can be used as an alternative when NOACs are contra-indicated or with a clear, pre-existing indication for VKAs*.

*ASA, acetyl salicylic acid; DAPT, dual antiplatelet therapy; INR, International Normalized ratio; NOAC, non-vitamin K oral anticoagulant; OAC, oral anticoagulation; TAVI, transcatheter aortic valve implantation; VKA, vitamin K antagonist*.

### Evolution of Prosthetic Valve Implantation and Current Numbers at the University Hospitals Leuven

**Figure 1:**
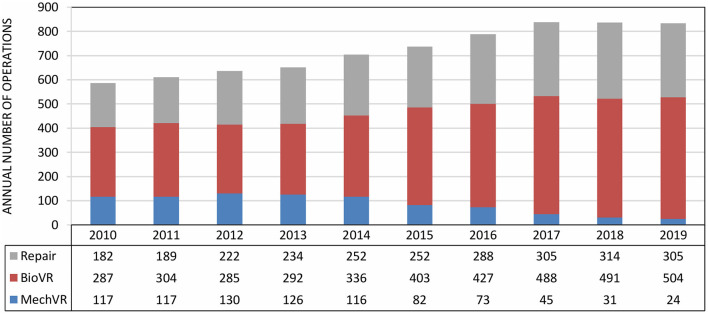
Evolution of surgical valve repair and replacement at the University Hospitals Leuven from 2010 to 2019. The annual numbers include data from all four heart valve positions. From 2010 to 2019, there has been a steady increase in the annual number of surgical valve repairs and bioprosthetic valve replacements, but a decrease in the annual number of mechanical valve implantations. BioVR, biological valve replacement (including Ross procedure); MechVR, mechanical valve replacement.

Despite significant evolutions in the field of prosthetic heart valves, surgical valve repair remains the first-choice surgical treatment for severe valvular heart disease when technically feasible. Over the past decade, there has been a steady increase in the annual number of patients undergoing surgical valve repair at the University Hospitals Leuven (Figure 1). In 2019, a total of 305 valves were surgically repaired, which included 20 aortic valves, 184 mitral valves and 101 tricuspid valves, and represent more than one third of all surgical valve interventions (*n* = 833, Table 3).

**Table 3:** Overview of surgical valve repair and replacement at the University Hospitals Leuven in 2019.

	**Aortic** ^**b**^	**Mitral** ^**c**^	**Tricuspid** ^**d**^	**Total**
Surgical valve repair	20 (2.4%)	184 (22.1%)	101 (12.1%)	305 (36.6%)
Bioprosthetic valve replacement	434 (52.1%) ^a^	65 (7.8%)	5 (0.6%)	504 (60.5 %)
Mechanical valve replacement	10 (1.2%)	14 (1.7%)	0	24 (2.9%)
Total	464 (55.7%)	263 (31.6%)	106 (12.7%)	833

*Numbers presented as “Absolute number of procedures (percentage of total procedures)”*

a *Including Ross procedure*.

b *Aortic valve procedures: 4.3% valve repair, 93.5% bioprosthetic valve replacement, 2.2% mechanical valve replacement*.

c *Mitral valve procedures: 70% valve repair, 24.7% bioprosthetic valve replacement, 5.3% mechanical valve replacement*.

d *Tricuspid valve procedures: 95.3% valve repair, 4.7% bioprosthetic valve replacement*.

However, when heart valve repair is not feasible, valve replacement is needed. Over the last decade, the ratio of mechanical and biological valves has changed considerably (Figure 1). The improved durability of new surgical bioprosthetic valves and the freedom from lifelong OAC led cardiac surgeons to prefer bioprostheses over mechanical valves. In addition, TAVI is increasingly performed because of growing expertise and the shift to a lower risk population. Today at the University Hospitals Leuven, about 85% of all valve replacement procedures are performed by cardiac surgery. In the remaining 15%, transcatheter techniques are used. In 2019, 504 bioprosthetic valves were surgically implanted of which 434 were in aortic position, including the Ross procedure, and 65 in mitral position (Table 3). Another 24 diseased valves were replaced by a mechanical prosthesis, including 10 aortic valves and 14 mitral valves.

### Antithrombotic Treatment for Mechanical Valves at the University Hospitals Leuven

All mechanical valves that are currently being implanted at the University Hospitals Leuven are bileaflet valves. After aortic valve replacement the standard target INR ranges from 2.0 to 2.5. In patients with an On-X aortic valve, a lower target INR of 1.5 to 2.0 in combination with low-dose aspirin can be considered from 3 months after surgery. For the mitral and tricuspid position an INR of 2.5 to 3.0 is targeted. In the presence of thromboembolic risk factors an increase of the target INR range by 0.5 can be considered: INR 2.5 to 3.0 for aortic prostheses and INR 3.0 to 3.5 for mitral and tricuspid prostheses. In case of thromboembolic events under standard VKA therapy, low-dose aspirin is associated. In patients with chronic coronary syndrome or atherosclerotic disease, we do not recommend the systematic addition of aspirin because the benefit has not been clearly demonstrated and concerns about an increased risk of bleeding remain.

Outside the target INR range, regarded as the optimal level of anticoagulation, we accept a wider window of INR values. The acceptable INR window is independent of thromboembolic risk factors and ranges from 1.8 to 3.0 for the aortic position and from 2.0 to 3.5 for the mitral and tricuspid position. The rational for this approach is to prevent an excessive number of adjustments of VKAs which have a negative impact on the time in the therapeutic range. Education of patients and physicians has proven to be paramount to reduce INR instability and variability, which is associated with more thromboembolic and bleeding complications and poor patient outcome (56). In addition, although not reimbursed in Belgium, we recommend INR self-management in well-selected patients given its clear added value in achieving an optimal INR control (18, 19).

Finally, few patients remain with older generation mechanical valves (e.g., Starr-Edwards, Björk-Shiley). These valves are no longer implanted nowadays, but given their thrombogenicity, a higher INR is targeted, and antithrombotic management needs to be individualized.

### Antithrombotic Treatment for Bioprosthetic Valves at the University Hospitals Leuven

For patients with a bioprosthetic aortic valve and no indication for OAC, lifelong low-dose aspirin is prescribed. As the mitral and tricuspid valve are more thrombogenic and the mitral valve population is on average older with numerous co-morbidities, oral anticoagulation is recommended during the early post-operative phase. In patients without indication for OAC, VKAs (INR 2.0 to 3.0) remain the first-choice anticoagulant therapy during the first 3 months after bioprosthetic mitral or tricuspid valve implantation, followed by lifelong low-dose aspirin. NOACs may be occasionally considered as an alternative, although not reimbursed in this context.

In patients with a bioprosthetic valve and no OAC indication, lifelong aspirin is recommended after the early post-operative phase. However, in patients at high bleeding risk, discontinuation of aspirin may be considered from 3 months after surgery.

With an indication for OAC, anticoagulant therapy is continued after bioprosthetic valve replacement, without adding aspirin. In this context, NOACs are preferred to VKAs and can be used in the early post-operative phase. Apixaban (5 mg or 2.5 mg, twice daily) and rivaroxaban (20 mg, once daily) are the NOACs that have been best investigated so far in the context of bioprosthetic valves (33, 57). VKAs are only appropriate in patients with a clear, pre-existing indication for VKAs and provided that INR monitoring is performed adequately.

### Antithrombotic Treatment for Surgical Valve Repair at the University Hospitals Leuven

Surgically repaired valves are much less thrombogenic compared to surgical valve replacement. Aortic valve repair is technically demanding and is mostly performed in expert centers. The population is young with few co-morbidities. In patients without OAC indication, low-dose aspirin is recommended during the first 3 months postoperatively. With an indication for OAC, oral anticoagulants are continued. NOACs are the first choice unless there is a clear, pre-existing indication for VKAs.

After mitral and tricuspid valve repair lifelong low-dose aspirin is recommended if there is no indication for OAC. In case of the need for anticoagulant therapy, the indication hereto and the patient's characteristics will guide the anticoagulant therapy. In accordance with current guidelines, many centers worldwide systematically prescribe anticoagulants after mitral and tricuspid valve repair. However, at the University Hospitals Leuven, thromboprophylaxis with lifelong aspirin monotherapy in patients with mitral and tricuspid valve repair without OAC indication has been routine practice for more than 20 years, which is in line with several observational studies reporting a similar thromboembolic risk between VKAs and low-dose aspirin after mitral valve repair (37, 38). Hence, anticoagulation is not routinely recommended, and such a strategy minimizes the risk of anticoagulation-related bleeding complications. With an indication for OAC after surgical mitral and tricuspid valve repair, VKAs may be used as an alternative to NOACs, although not routinely used given the need for routine INR monitoring and the high INR variability.

### Antithrombotic Treatment for Transcatheter Valve Procedures at the University Hospitals Leuven

The recommendations for antithrombotic therapy after TAVI are mainly based on the POPular-TAVI trial (46, 50). For TAVI patients without a pre-existing indication for OAC, lifelong aspirin monotherapy is prescribed, unless DAPT is indicated for another reason (e.g., recent stenting). For TAVI patients with an indication for OAC, we prefer NOACs to VKAs (INR 2.0–3-0), without association of anti-platelet agents, unless they are indicated for another reason.

After percutaneous edge-to-edge repair with MitraClip™, patients without OAC indication will receive DAPT, with aspirin and clopidogrel, for 1 month, followed by lifelong low-dose aspirin. In patients with an indication for chronic OAC, OAC is continued, but aspirin is systematically added during the 1st month after the procedure. NOACs are the first choice unless there is a specific indication for VKAs.

Finally, a very limited number of patients undergo transcatheter mitral or tricuspid valve replacement in a dysfunctional native or bioprosthetic valve or ring annuloplasty (not included in Table 2). These are emerging techniques and consequently, the antithrombotic management has not yet been extensively studied. In our center, all these patients will receive OAC with VKAs for a minimum of 6 months after the intervention. After this period, NOACs can be used as an alternative to VKAs in case of unstable INR control or bleeding complications. Otherwise, VKAs can be continued.

### Antithrombotic Therapy in Patients Requiring Combined Anticoagulant and Antiplatelet Therapy

In some situations, patients with need for OAC in the context of a prosthetic valve can have a concomitant indication for anti-platelet therapy, such as an acute coronary syndrome (ACS), recent percutaneous coronary intervention (PCI) or concomitant coronary artery bypass graft (CABG).

A significant proportion of patients undergo CABG at the time of valve surgery. After CABG for chronic coronary syndrome, lifelong low-dose aspirin is recommended (58, 59). In patients with an indication for OAC undergoing combined CABG and valve surgery, low-dose aspirin is associated during the first 3 months to secure graft patency but can be discontinued thereafter.

In the context of an ACS or recent PCI, DAPT with aspirin and clopidogrel is indicated for 6 to 12 months (58, 59). However, the patient group with a pre-existing indication for OAC poses a clinical challenge as the combination of an oral anticoagulant with DAPT can significantly increase the risk of bleeding. For years, the dogma of triple antithrombotic therapy, including OAC and DAPT, has persisted. However, in the context of atrial fibrillation, it has now been clearly demonstrated that dual antithrombotic therapy with an oral anticoagulant and a P2Y_12_ inhibitor results in less bleeding compared to triple therapy (60). Furthermore, in terms of bleeding, dual therapy including a NOAC is superior to therapy including a VKA (60). However, similar studies in patients with prosthetic heart valves are lacking and NOACs are often contra-indicated. The WOEST trial is the only trial in this context which included patients with a mechanical valve and also argues in favor of dual therapy (61). By analogy with atrial fibrillation, a dual antithrombotic strategy with an OAC and clopidogrel for 6 to 12 months is suggested in patients requiring OAC in the context of a prosthetic valve and an ACS or recent PCI (58). However, when the risk of thromboembolism outweighs the risk of bleeding, a short period (maximum 1 month) of triple therapy can be considered (62). Vice versa, in patients with high bleeding risk or frailty, shorter dual therapy regimens might be warranted.

### Unsolved Questions and Starting Points for Research

- Evaluating the efficacy and safety of factor Xa inhibitors in patients with a mechanical valve.
- Evaluating the efficacy and safety of NOACs in the early post-operative phase after bioprosthetic mitral and tricuspid valve replacement in patients without indication for oral anticoagulation.
- Evaluating the benefit-risk ratio of lifelong aspirin administration in patients with a surgical bioprosthetic valve and no indication for oral anticoagulation.
- Further clarifying the use of NOACs after surgical valve repair.
- Evaluating the optimal antithrombotic regimen after percutaneous edge-to-edge mitral valve repair.
- Comparing dual antithrombotic therapy vs. triple therapy after an acute coronary syndrome or percutaneous coronary intervention, in patients receiving oral anticoagulation for a prosthetic heart valve.

## Conclusion

The heterogeneity between guidelines, the increasing diversity of prosthetic valves and antithrombotic drugs, and the sharp rise of minimally invasive transcatheter techniques make daily clinical decision-making on antithrombotic treatment after heart valve replacement or repair difficult and complex. After an analysis of the main guidelines and the available evidence in this area, and a multidisciplinary team discussion, involving more than 800 valve patients per year, a consensus practical guidance was formulated. This will serve from 2021 onwards as a standard guideline in our center and provide a clear recommendation for antithrombotic treatment after surgical or transcatheter heart valve intervention. Updates will be necessary in the future once new data become available.

## Data Availability Statement

The raw data supporting the conclusions of this article will be made available by the authors, without undue reservation.

## Author Contributions

AV reviewed the literature, wrote the first draft of the manuscript, designed the figures and tables and made adjustments according to the suggestions of the co-authors. PVerh supervised this paper, coordinated the multidisciplinary discussion, and critically reviewed the manuscript. M-CH, PVerb, CD, TV, and BM performed a detailed review and provided substantial insights. ML provided the surgical data of the University Hospitals Leuven. All authors participated in the multidisciplinary discussion, which resulted in the final consensus, contributed to the article, and approved the submitted version.

## Conflict of Interest

The authors declare that the research was conducted in the absence of any commercial or financial relationships that could be construed as a potential conflict of interest.

## Publisher's Note

All claims expressed in this article are solely those of the authors and do not necessarily represent those of their affiliated organizations, or those of the publisher, the editors and the reviewers. Any product that may be evaluated in this article, or claim that may be made by its manufacturer, is not guaranteed or endorsed by the publisher.
